# Hematometra presenting as an acute abdomen in a 13-year-old postmenarchal girl: a case report

**DOI:** 10.1186/1752-1947-6-419

**Published:** 2012-12-12

**Authors:** Peter Klimek, Miriam Klimek, Ulf Kessler, Valerie Oesch, Rainer Wolf, Enno Stranzinger, Michael D Mueller, Zacharias Zachariou

**Affiliations:** 1 Department of Pediatric Surgery, Inselspital, University of Berne, Berne, Switzerland; 2 Department of Obstetrics and Gynecology, Regionalspital Emmental, Burgdorf, Switzerland; 3 Department of Pediatric Surgery, Kantonsspital Aarau, Aarau, Switzerland; 4 Department of Obstetrics and Gynecology, Inselspital, University of Berne, Berne, Switzerland; 5 Department of Diagnostic, Interventional and Pediatric Radiology, Inselspital, University of Berne, Berne, Switzerland

**Keywords:** Adolescent, Acute abdomen, Genital malformation, Hematometra, Uterus didelphys

## Abstract

**Introduction:**

Most underlying diseases for abdominal pain in children are not dangerous. However some require rapid diagnosis and treatment, such as acute ovarian torsion or appendicitis. Since reaching a diagnosis can be difficult, and delayed treatment of potentially dangerous diseases might have significant consequences, exploratory laparoscopy is a diagnostic and therapeutic option for patients who have unclear and potentially hazardous abdominal diseases. Here we describe a case where the anomaly could not be identified using a laparoscopy in an adolescent girl with acute abdomen.

**Case presentation:**

A 13-year old postmenarchal caucasian female presented with an acute abdomen. Emergency sonography could not exclude ovarian torsion. Accurate diagnosis and treatment were achieved only after an initial laparoscopy followed by a laparotomy and after a magnetic resonance imaging scan a further laparotomy. The underlying disease was hematometra of the right uterine horn in a uterus didelphys in conjunction with an imperforate right cervix.

**Conclusion:**

This report demonstrates that the usual approach for patients with acute abdominal pain may not be sufficient in emergency situations.

## Introduction

Abdominal pain in children is one of the most common reasons for emergency evaluation. Underlying diseases often depend on the child’s age and are self limited minor conditions. However it is important to recognize serious diseases, which might require specific treatment, or invasive procedures such as acute ovarian torsion or appendicitis [1].

Ruling out serious surgical conditions can be difficult, especially in infants. Moreover, delay in the treatment of potentially dangerous surgical abdominal diseases might have significant consequences. Therefore, exploratory laparoscopy is a diagnostic and therapeutic option for patients with unclear and potentially hazardous abdominal diseases [2]. Nonetheless, performing exploratory laparoscopy on children with acute abdominal pain remains a controversial issue; and should only be performed after taking into account the risk/ benefit ratio and the surgeon’s experience [2].

Here we describe a case where the anomaly could not be identified using a laparoscopy in an adolescent girl with acute abdomen.

## Case presentation

A 13-year old caucasian female presented in the pediatric emergency unit with acute right-sided lower abdominal pain.

There were no signs of infectious, gastrointestinal or urinary problems. Menarche had started nine months previously with regular menstrual bleeding. However, the patient suffered from symptoms of primary dysmenorrhea. Her most recent menstruation had started three days before.

On examination the patient presented with severe tenderness in the lower abdomen. External genital inspection showed an intact hymen and menstrual discharge. Blood and urine tests were normal. Transabdominal ultrasound with a low-filled bladder detected an 8cm × 6.5cm hypoechoic mass in the right iliac fossa without perfusion, interpreted as a uterine malformation, a hematocolpos or an ovarian torsion.

Due to the risk of an underlying ovarian torsion, the patient was taken to theater for vaginoscopy under anesthesia and, in the absence of any pathologic findings, for exploratory laparoscopy and, if necessary, laparotomy. Vaginoscopy showed a normal vaginal vault and a single cervix. Ovarian torsion could not be excluded by laparoscopy due to right-sided peritubal adhesions. Following a Pfannenstiel laparotomy, a bicornuate uterus with a blood- filled right uterine horn was diagnosed. The ovaries and tubes were normal. The right uterine hematometra was transabdominally drained.

**Figure 1:**
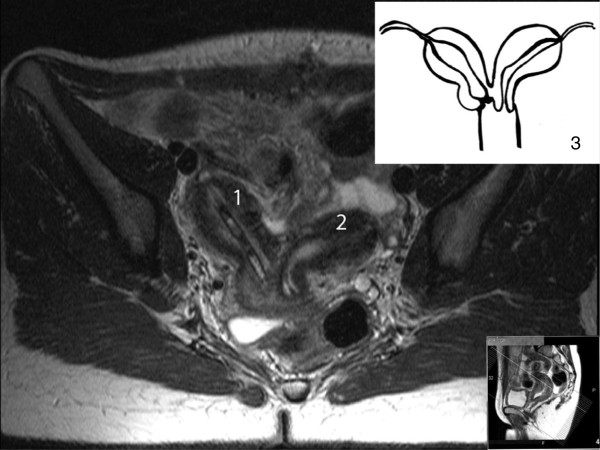
Magnetic resonance image T2-weighted; 1: right horn of the uterus with drainage tube in place, 2: Left horn of the uterus, 3: Illustration of the configuration of the uterus in the present case.

Postoperative magnetic resonance imaging (MRI) was performed to clearly visualize the anatomic condition. It confirmed two separate uterocervical cavities without communication of the right cervix to the vagina (Figure 1). Right-sided kidney aplasia was noted by ultrasound and MRI.

The continuity between the right uterus and the vagina was reconstructed one week later. The girl recovered fully and had normal menstruation without dysmenorrhea when we saw her at follow-up, six months later.

## Discussion

This patient illustrates the challenges of diagnosis and treatment of uterine malformations. The presence of acute abdominal pain required us to intervene immediately.

One reason why an internal genital malformation could not be diagnosed earlier were the anamnesis of menarchal onset with regular bleeding for nine months, as well as the physical examination showing severe tenderness in the lower abdomen together with a regular hymen.

Since the patient presented with an acute abdomen, and ovarian torsion was a likely possibility following transabdominal ultrasound, we took the patient to theater for vaginoscopy, laparoscopy and finally laparotomy.

Could more accurate diagnostic imaging have prevented the emergency intervention? We routinely use transabdominal ultrasound with, if possible, a fully distended urine bladder as first imaging choice for cases of unclear abdominal pain in girls [3]. Transvaginal ultrasound might have revealed the underlying disease in our patient, but is not recommended in non-sexually active girls.

MRI of the pelvis is the imaging of choice after ultrasound examination if uterine malformation is a possibility [4]. In our institution the access to abdominal MRI in emergency situations is limited.

For suspected ovarian torsion immediate care is required and surgical detorsion within four hours after onset of symptoms might reduces tissue damage [5,6]. Taken altogether, we believe that we made the correct decision.

How can renal agenesis be interpreted in the present case? Uterus didelphys is caused by an incomplete fusion of the Mullerian duct system. In 36% of all cases, Mullerian anomalies are associated with other structural abnormalities; most of them being renal malformations because of the concomitant embryologic development [7].

The most widely-accepted classification of uterine malformations from the American Fertility Society, does not take into account the frequently associated vaginal, cervical or urological anomalies [8-10]. The renal and genital anomalies found in our patient could be more precisely described by another classification from Oppelt et al. as *V0 C2a U2 A0 MR*[11].

## Conclusion

This report demonstrates the challenges and pitfalls in diagnosis and treatment of postmenarchal girls with acute abdominal pain, and shows that the usual approach to patients with acute abdominal pain may not be sufficient in emergency situations. Nonetheless diagnostic laparoscopy remains the standard emergency approach for patients with potentially hazardous acute abdominal pain. Judicious use of MRI in adolescent girls may help to obviate the need for transvaginal ultrasonography in circumstances where the diagnosis is difficult to establish.

## Consent

Written informed consent was obtained from the patient’s legal guardian for publication of this manuscript and accompanying images. A copy of the written consent is available for review by the Editor-in-Chief of this journal.

## Competing interests

The authors declare that they have no competing interests.

## Authors’ contributions

All authors analyzed and interpreted the patient history, data, images and clinical findings. PK, MK, and UK wrote the manuscript. All authors read and approved the final manuscript.
